# Not all viruses cause disease: HERV-K(HML-2) in healthy human tissues

**DOI:** 10.1371/journal.pbio.3001884

**Published:** 2022-10-31

**Authors:** Smitha Srinivasachar Badarinarayan, Daniel Sauter

**Affiliations:** 1 Department of Molecular Biology and Genetics, Cornell University, Ithaca, New York, United States of America; 2 Institute for Medical Virology and Epidemiology of Viral Diseases, University Hospital Tübingen, Tübingen, Germany

## Abstract

Human endogenous retroviruses (HERVs) make up a significant part of our genomes. Their expression is frequently associated with disease, but this Primer explores the implications of a new study in PLOS Biology which found that HERV-K (HML-2) is expressed in more than 50 healthy tissues. Several of these viral DNA fossils have retained intact open reading frames and may encode functional proteins.

## Teaser

Human endogenous retroviruses (HERVs) make up a significant part of our genome. While their expression is frequently associated with disease, a new study in *PLOS Biology* found that HERV-K(HML-2) is expressed in more than 50 healthy tissues.

Viruses are everywhere. They infect virtually all organisms on this planet. They can be found in the air we breathe, the flowers in our garden, and the depths of the ocean. Some of them can even be found in our DNA. These so-called human endogenous retroviruses (HERVs) represent remnants of once-infectious viruses that became fixed in our genome. While some of them still have the potential to produce virus-like particles, HERVs have lost their ability to generate infectious virions that are transmitted horizontally. Instead, they persist in our DNA as viral fossils that are passed on vertically from generation to generation.

Viruses are generally perceived as nasty pathogens, whose transmission and spread must be prevented. Even the infectious ancestors of endogenous retroviruses may have been pathogenic, and expression of HERVs such as HERV-K(HML-2) has been associated with cancer, neurological disorders, and other diseases. However, several HERVs have also been co-opted during evolution and exert important physiological functions. The prime examples are 2 virus-derived glycoproteins (Syncytin-1 and Syncytin-2) that mediate cell–cell fusion during placenta development [1]. Without these viral proteins, normal pregnancy would not be possible. Other HERVs regulate cellular gene expression. For example, alcohol digestion is modulated by a virus-derived *cis*-regulatory element enhancing expression of the alcohol dehydrogenase 1C gene (*ADH1C*) [2]. Some HERVs even contribute to antiviral immune responses, e.g., by regulating antiviral gene expression [3,4].

**Fig 1:**
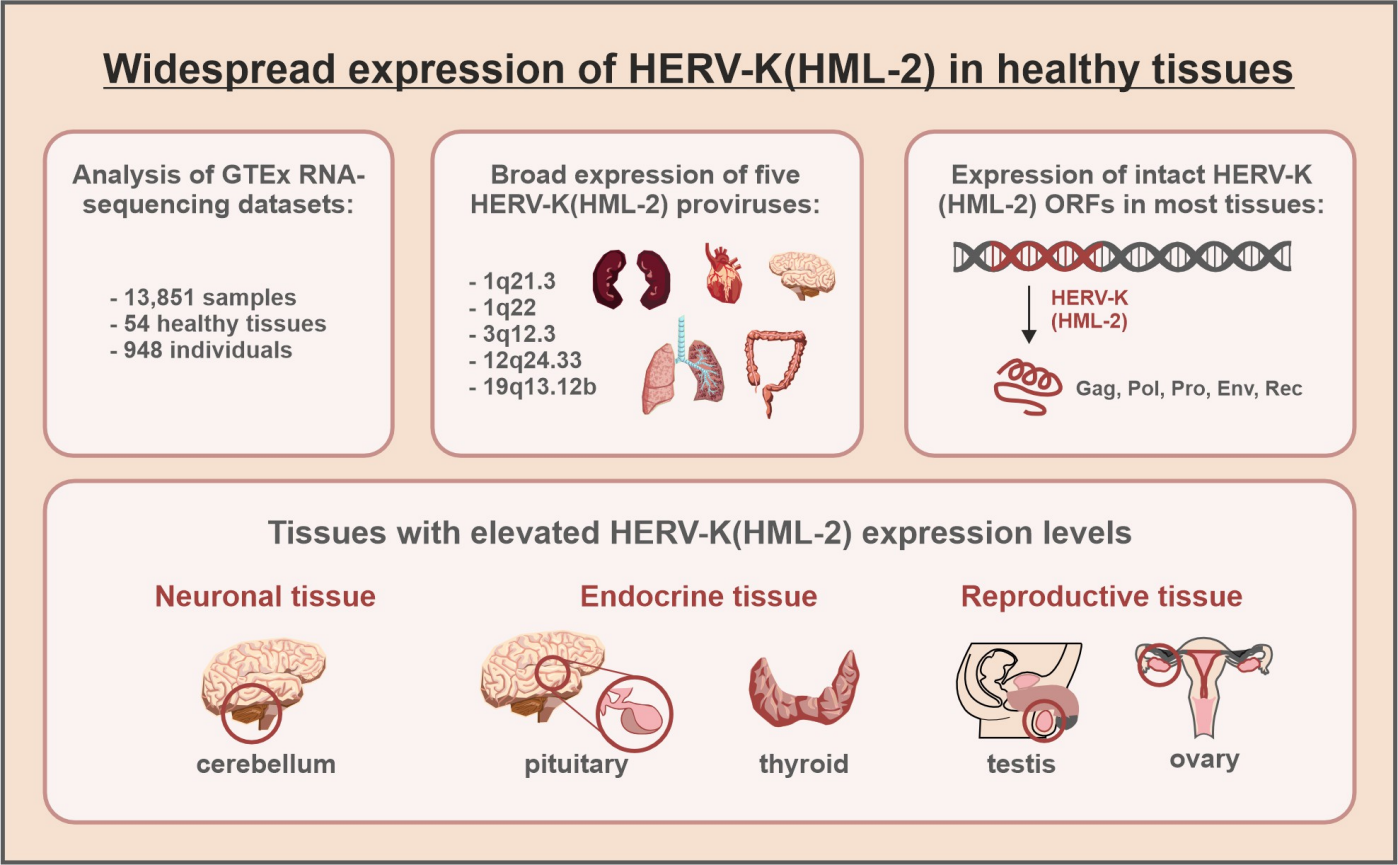
Endogenous retroviruses of the HERV-K(HML-2) subclade are broadly expressed in healthy human tissues. About 8% of the human genome consist of HERVs. By analysing the transcriptomes of more than 13,000 human samples, Burn and colleagues show that HERV-K(HML-2), the evolutionarily youngest ERV subclade in humans, is broadly expressed in healthy cells and tissues. Particularly high HERV-K(HML-2) transcript levels were observed in the cerebellum, pituitary, thyroid, and reproductive tissues. Five HERV-K(HML-2) proviruses (located at 1q21.3, 1q22, 3q12.3, 12q24.33, and 19q13.12b) were expressed in almost every tissue. The authors did not observe any association of HERV-K(HML-2) expression with age and only a minor association with sex in some tissues (e.g., breast). However, evolutionarily older HERV-K(HML-2) proviruses tend to be expressed to higher levels than their younger counterparts. Intriguingly, some of the expressed HERV-K(HML-2) repeats harbour intact open reading frames that may encode for Gag, Pro, Pol, Env, or Rec proteins in several healthy tissues.

To better understand the role of HERVs in human health and disease, a systematic comparison of their activity in healthy versus diseased tissues is required. Most previous studies, however, have focussed on HERV activation in the context of disease, and there is limited information on HERV expression in non-diseased tissues, particularly at the level of individual HERV loci. In this issue, Burn and colleagues closed this gap by systematically analysing HERV transcription in more than 50 healthy tissues from almost 1,000 donors [5] (Fig 1). The authors took advantage of RNA-sequencing datasets available via the Genotype Tissue and Expression (GTEx) Project [6]. For their analyses, Burn and colleagues focused on HERV-K(HML2). This ERV subclade represents the youngest HERV group and includes proviruses that are unique to humans. Some HERV-K(HML-2) proviruses are even polymorphic in the human population and may contribute to inter-individual variation [7]. Like all HERVs, HERV-K(HML-2) insertions have accumulated numerous deletions and mutations. Still, some HERV-K(HML-2) elements have retained intact open reading frames (ORFs) that may encode for functional proteins.

Intriguingly, HERV-K(HML-2) expression was detectable in all tissues examined. Particularly, high expression levels were observed in the brain (cerebellum and pituitary), thyroid, and reproductive tissues (testes and ovaries) (Fig 1). The high levels of HERV-K(HML-2) transcripts in reproductive tissues may be a consequence of their ancestor’s ability to infect germ cells. To establish themselves in the human genome, retroviruses must enter the germ line. Thus, HERV-K(HML-2) has evolved from an exogenous retrovirus that infected germ cells and most likely exploited cellular transcription factors that are highly active in reproductive tissues. These properties may allow efficient LTR-mediated HERV-K(HML-2) transcription in testes and ovaries today. It is tempting to speculate that expression of HERV-K(HML-2) in other tissues may also be a relic of the tissue tropism of the ancestral exogenous retrovirus. Alternatively, selection processes may have changed the expression patterns of HERV-K(HML-2) after their fixation in the host genome.

Importantly, Burn and colleagues did not only analyse total HERV-K(HML-2) transcription, but also expression of individual HERV-K(HML-2) loci. They detected 37 HERV-K(HML-2) proviruses that are expressed in at least 1 tissue. Five of them (1q21.3, 1q22, 3q12.3, 12q24.33, and 19q13.12b) were broadly transcribed in almost every tissue (Fig 1).

Intriguingly, some of the HERV-K(HML-2) loci expressed in healthy tissues also contain intact ORFs (Fig 1). For example, *gag*-derived proteins may be produced by the broadly expressed HERV-K(HML-2) locus 3q12.3 and/or 12q14.1, which is specifically expressed in kidneys. Although stability and potential functions of these Gag proteins remain to be determined, HERV-derived Gag proteins may negatively interfere with the assembly of exogenous retroviruses [8]. In addition to Gag, a functional Env protein expressed from HERV-K(HML-2) 7p22.1a may be present in several healthy tissues, including blood. This particular Env protein has previously been shown to retain its fusogenic activity [9] and may thus induce syncytia formation in different tissues, provided that its receptor is also present. Notably, HERV-K(HML-2) 7p22.1a Env was also suggested to reduce HIV-1 infection [10]. Besides *gag* and *env* genes, intact HERV-K(HML-2) *rec* ORFs were also shown to be expressed in various tissues, confirming and expanding findings of an earlier study [11]. HERV-K(HML-2) Rec can be considered a functional homolog of HIV Rev. Both proteins interact with RNA structures and mediate the export of unspliced or incompletely spliced (viral) mRNA from the nucleus to the cytoplasm. While the presence of *rec* transcripts in several healthy tissues suggests that Rec proteins may have been co-opted by the human organism, the exact function of Rec proteins in human cells remains to be determined.

In summary, the transcriptome analyses by Burn and colleagues help to identify active HERV loci that are neither cause nor consequence of disease, but may have neutral or even beneficial effects. Their study provides a valuable dataset of healthy controls that can be used to better assess the relevance of HERV activation in aberrant cells. Notably, the identification of HERV-K(HML-2) ORFs strongly suggests that virus-derived proteins are normal components of the human proteome in many tissues. Together with proteomic analyses, the findings by Burn and colleagues will therefore also help to assess the suitability of HERV proteins as biomarkers and their specificity for certain cancer entities and other aberrant cells.

It should be borne in mind that the HERV-K(HML-2) group analysed in the present study is only one of many HERV subclades. It remains to be determined whether other HERV members exhibit similarly broad expression in healthy cells and tissues. Moreover, HERVs can also be active without being transcribed, e.g., by acting as enhancers or suppressors of gene expression. Thus, the number of HERVs that are active in our cells is most likely higher than we think, and many physiological HERV functions remain to be discovered.
